# Combined QTL mapping, GWAS and transcriptomic analysis revealed a candidate gene associated with the timing of spring bud flush in tea plant (*Camellia sinensis*)

**DOI:** 10.1093/hr/uhad149

**Published:** 2023-07-26

**Authors:** Yujie Liu, Si Chen, Chenkai Jiang, Haoran Liu, Junyu Wang, Weizhong He, Doogyung Moon, Jiedan Chen, Liang Chen, Jianqiang Ma

**Affiliations:** Key Laboratory of Biology, Genetics and Breeding of Special Economic Animals and Plants, Ministry of Agriculture and Rural Affairs, Tea Research Institute of the Chinese Academy of Agricultural Sciences, Hangzhou 310008, China; Key Laboratory of Biology, Genetics and Breeding of Special Economic Animals and Plants, Ministry of Agriculture and Rural Affairs, Tea Research Institute of the Chinese Academy of Agricultural Sciences, Hangzhou 310008, China; Institute of Sericulture and Tea, Zhejiang Academy of Agricultural Sciences, Hangzhou 310021, China; Key Laboratory of Biology, Genetics and Breeding of Special Economic Animals and Plants, Ministry of Agriculture and Rural Affairs, Tea Research Institute of the Chinese Academy of Agricultural Sciences, Hangzhou 310008, China; Key Laboratory of Biology, Genetics and Breeding of Special Economic Animals and Plants, Ministry of Agriculture and Rural Affairs, Tea Research Institute of the Chinese Academy of Agricultural Sciences, Hangzhou 310008, China; Tea Research Institute, Lishui Academy of Agricultural and Forestry Sciences, Lishui 323000, China; Research Institute of Climate Change and Agriculture, National Institute of Horticultural and Herbal Science, Jeju 690-150, Korea; Key Laboratory of Biology, Genetics and Breeding of Special Economic Animals and Plants, Ministry of Agriculture and Rural Affairs, Tea Research Institute of the Chinese Academy of Agricultural Sciences, Hangzhou 310008, China; Key Laboratory of Biology, Genetics and Breeding of Special Economic Animals and Plants, Ministry of Agriculture and Rural Affairs, Tea Research Institute of the Chinese Academy of Agricultural Sciences, Hangzhou 310008, China; Key Laboratory of Biology, Genetics and Breeding of Special Economic Animals and Plants, Ministry of Agriculture and Rural Affairs, Tea Research Institute of the Chinese Academy of Agricultural Sciences, Hangzhou 310008, China

Dear Editor,

The timing of the spring bud flush (TBF) is a crucial agronomic trait for the tea plant, as it strongly influences the yield and economic value of harvested fresh tea leaves. The TBF of tea plant is generally defined as the date when >30% of the growing tender shoots have reached the stage of one bud with one to three leaves, referred as to the stage of one and a bud, two and a bud, and three and a bud, respectively. The TBF is a complex phenotype controlled by quantitative trait loci (QTL), which have previously been identified from several populations [1, 2]. However, the candidate regions and underlying genes for these QTL remain difficult to ascertain through further fine mapping, due to the lack of appropriate biparental populations, which has been frustrated by the nature of the long juvenile phase and self-incompatibility in the tea plant. Since the release of accurate reference genomes and large-scale genome resequencing data of tea plant germplasms [3, 4] it has become possible to use genome-wide association study (GWAS) and omics approaches to facilitate revealing the genetic basis of target traits.

In our present study, a major QTL for the TBF trait (the stage of one and a bud), named *qTBF4-1*, was identified based on an *F*_1_ mapping population composed of 183 three-year-old individuals derived from the crossing of early-sprouting cultivar ‘Yingshuang’ and the late-sprouting cultivar ‘Beiyue Danzhu’ (Supplementary Data Tables S1 and S2). QTL mapping was performed using the Multiple QTL Mapping (MQM) method of MapQTL 6 software, by means of which the 95% genome-wide LOD significance thresholds (LOD > 3.0) were determined by permutation tests. *qTBF4-1* was located on chromosome 4 (Chr4) in the interval of 182.143–189.369 Mb, and explained 16.4% of the phenotypic variation on average (Fig. 1A). Comparative genome analysis showed that the position of *qTBF4-1* overlapped with that of previously reported *qSPI4* (in the interval of 157.841–196.299 Mb) [2], indicating that this QTL was stable under different genetic backgrounds and environments, and could play an important role in regulating the TBF of tea plant.

**Figure 1:**
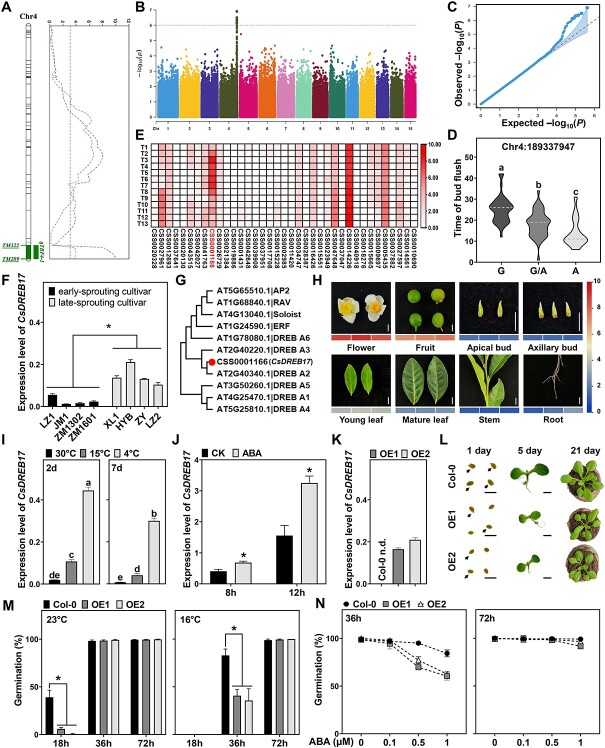
Identification and characterization of *CsDREB17* as a candidate gene underlying the major QTL associated with the timing of spring bud flush (TBF) in tea plant. **A** A major QTL, named *qTBF4-1*, related to the TBF on chromosome 4 (Chr4) with an interval of 7.226 Mb, was detected across 2 years by linkage mapping. **B** Manhattan plot for the GWAS analysis revealing a significant SNP peak co-located with *qTBF4-1* on Chr4. The line is the threshold level (−log_10_*P* = 6). **C** QQ plot of the GWAS analysis. **D** Violin plot of TBF values among the test tea accessions when stratified by genotype at the lead GWAS SNP (Chr4:189337947). **E** Expression patterns of 37 genes within the QTL candidate region identified by RNA-seq during spring bud flush, including 13 time points. **F** Expression levels of candidate gene *CsDREB17* in different tea cultivars with distinct TBF. **G** Phylogenetic analysis of *CsDREB17* with representative *AP2/ERF* subfamily genes in *Arabidopsis thaliana*. **H** Tissue-specific expression patterns of *CsDREB17*. Expression level was determined by qRT–PCR. **I**, **J** Expression patterns of *CsDREB17* in tea plant under different temperature conditions after 2 and 7 days (**I**) and under 50 mg/l exogenous ABA treatment after 8 and 12 h (**J**). **K** Expression levels of *CsDREB17* in *T*_3_-generation homozygous lines of overexpressing *CsDREB17* (OE1 and OE2) and wild-type (Col-0) *Arabidopsis* plants. **L** Phenotype of transgenic and wild-type *Arabidopsis* grown at 23°C for 1, 5, and 21 days. Scale bar = 1 mm. **M, N** Germination rates of Col-0, OE1, and OE2 under normal temperature (23°C) and chilling temperature (16°C) (**M**) and under exogenous ABA treatment at various concentrations (**N**). Three independent biological replicates were performed for each experiment. At least 50 seeds of every line were used for each replicate. The letters above columns and asterisks in panels D, F, I, J and M represent the significance of the differences.

For fine mapping of the QTL, we performed a GWAS analysis using a diversity panel of 115 genotyped tea accessions (Supplementary Data Table S3) from TeaGVD [4]. The kinship matrix and association analysis were carried out by EMMAX (Efficient Mixed-Model Association eXpedited). At a significance level of *P* < 1 × 10^−6^, six SNPs were identified as being associated with the TBF (Supplementary Data Table S4), and the Manhattan plot showed a significant peak co-located with *qTBF4-1* on Chr4 (Fig. 1B). The quantile–quantile (QQ) plot implied that the population structure and kinship relationship were well controlled in the GWAS (Fig. 1C). The phenotypic values of TBF among the above-mentioned tea accessions could be clearly distinguished when stratified by genotype at the lead SNP (Chr4:189337947) (Fig. 1D). To validate the accuracy of genotypes, we detected 16 SNPs close to the QTL region among 14 tea accessions by KASP assay. The results showed that the consistent rates of the genotyped SNPs of TeaGVD and KASP ranged from 85.7 to 92.9% with an average of 91.3%, suggesting that the genotypic data from TeaGVD were reliable (Supplementary Data Tables S3 and S5). Thus, combined QTL mapping and GWAS analysis narrowed down the locus to the interval of 188.549 and 189.369 Mb, within which a total of 37 annotated genes were identified.

We subsequently investigated the dynamic transcriptome of tea plant cultivar ‘Longjing 43’ (hereafter referred to as ‘LJ43’) during the spring bud flush, including 13 time points. A candidate gene (CSS0001166) was screened out from the above 37 genes by expression pattern analysis. CSS0001166 was actively expressed in the dormant bud, whereas its expression level sharply decreased at the beginning of bud flush (Fig. 1E). Additionally, we examined the expression of CSS0001166 in spring apical buds of cultivars with markedly different TBF. The results showed that expression levels of CSS0001166 were significantly higher in late-sprouting cultivars than in early-sprouting cultivars (Fig. 1F). Therefore, we propose the CSS0001166 is the most likely candidate gene for *qTBF4-1*.

Sequence analysis indicated that CSS0001166 belonged to the AP2/ERF transcription factor family, and it was classified into the A2 group of the DREB subfamily according to the results of phylogenetic analysis (Fig. 1G). Thus, we named it *CsDREB17*. The tissue-specific expression in the cultivar ‘LJ43’ showed that the expression level of *CsDREB17* was highest in the flower and fruit, followed by the young leaf, mature leaf, root, apical bud, axillary bud, and stem (Fig. 1H). As temperature and ABA are the two main factors that influence bud break in perennial plants [5, 6], we evaluated expression levels of *CsDREB17* in tea plant under different temperature conditions and exogenous ABA treatment. The results revealed that exposure to lower temperature and ABA dramatically induced the expression of *CsDREB17* (Fig. 1I and J).

Although the bud flush of perennials and seed germination of *Arabidopsis* were different physiological processes, a similar pathway sharing homologous regulatory genes has been identified [7, 8]. We therefore overexpressed *CsDREB17* in *Arabidopsis* to explore its potential function since genetic transformation remains a bottleneck in tea plant. The CDS of *CsDREB17* was cloned into a pK7FWG2.0 (35S promoter) vector to construct transgenic *Arabidopsis*. Two *T*_3_-generation homozygous lines (OE1 and OE2) with high expression levels of *CsDREB17* were generated for further experiments together with the wild type (Col-0) (Fig. 1K). The overexpression lines exhibited delayed germination and minor growth retardation relative to the wild type, even though they had fairly similar survival rates (Fig. 1L). Only 5.9% (OE1) and 0.6% (OE2) of the seeds germinated compared with 39.1% in Col-0 18 h after planting under the normal growth condition (23°C) (Fig. 1M). Furthermore, germination rates after 36 h were remarkably reduced in the overexpression lines when grown at 16°C (Fig. 1M) or when fed ABA concentrations >0.5 μM. Compared with the wild type, the average germination rates of the overexpression lines decreased by 21.6 and 22.9% under 0.5 and 1 μM ABA concentrations, respectively (Fig. 1N). Collectively, our data demonstrated that overexpression of *CsDREB17* in *Arabidopsis* led to delayed germination and enhanced chilling and ABA sensitivity during germination. This is consistent with the previous findings for the homologue of *DREB2C* (AT2G40340) in *Arabidopsis* [9, 10].

In brief, we identified a candidate gene underlying the major QTL *qTBF4-1* associated with the TBF in tea plant by integrating QTL mapping, GWAS, and transcriptomic analysis. The candidate gene, named *CsDREB17*, encodes an AP2/ERF transcription factor, which was confirmed based on gene function annotation, haplotype analysis, and expression analysis in different cultivars with distinct TBF. The expression of *CsDREB17* was chilling- and ABA- inducible in tea plant. Overexpression of *CsDREB17* caused delayed germination under the normal growth condition and chilling or ABA treatments in transgenic *Arabidopsis*. Taken together, these results suggested that *CsDREB17* possibly acts as a negative regulator of spring bud flush in tea plant. This study provides a genetic basis for further work to decipher the mechanism controlling the TBF in tea plant.

## Acknowledgements

This work was supported by grants from National Key Research and Development Program of China (2021YFD1200200), the Major Project of Agricultural Science and Technology in Breeding of Tea Plant Variety in Zhejiang Province (2021C02067), the Chinese Academy of Agricultural Sciences through the Agricultural Science and Technology Innovation Program (CAASASTIP-2017-TRICAAS), the Earmarked Fund for China Agriculture Research System of MOF and MARA (CARS-19), and the National Natural Science Foundation of China (U22A20500, 32202553).

## Author contributions

J.M. and L.C. conceived the study and designed the experiments. Y.L., S.C., C.J., H.L., J.W., W.H., and D.M. performed the experiments. Y.L., S.C., J.C., and J.M. analyzed the data and organized the figures. Y.L. and J.M. wrote the manuscript. All authors reviewed and approved the final manuscript.

## Data availability

The RNA-seq data of tea plant during spring bud flush can be found in NCBI with the accession number PRJNA898859. The other relevant data can be found within the manuscript and its supplementary information.

## Conflict of interest

All authors declare that they have no conflict of interest.

## Supplementary data

Supplementary data is available at *Horticulture Research* online.

## Supplementary Material

Web_Material_uhad149
